# The mRNA dynamics underpinning translational control mechanisms of *Drosophila melanogaster* oogenesis

**DOI:** 10.1042/BST20231293

**Published:** 2024-09-12

**Authors:** Livia V. Bayer, Samantha N. Milano, Diana P. Bratu

**Affiliations:** 1 Department of Biological Sciences, Hunter College, City University of New York, New York, NY 10065, USA; 2 Program in Molecular, Cellular and Developmental Biology, The Graduate Center, City University of New York, New York, NY 10016, USA

**Keywords:** condensates, *Drosophila* oogenesis, mRNA translational control, *oskar*, P bodies, post transcriptional gene expression regulation

## Abstract

Advances in the study of mRNAs have yielded major new insights into post-transcriptional control of gene expression. Focus on the spatial regulation of mRNAs in highly polarized cells has demonstrated that mRNAs translocate through cells as mRNA:protein granules (mRNPs). These complex self-assemblies containing nuclear and cytoplasmic proteins are fundamental to the coordinated translation throughout cellular development. Initial studies on translational control necessitated fixed tissue, but the last 30 years have sparked innovative live-cell studies in several cell types to deliver a far more nuanced picture of how mRNA-protein dynamics exert translational control. In this review, we weave together the events that underpin mRNA processes and showcase the pivotal studies that revealed how a multitude of protein factors engage with a transcript. We highlight a mRNA's ability to act as a ‘super scaffold’ to facilitate molecular condensate formation and further moderate translational control. We focus on the *Drosophila melanogaster* germline due to the extensive post-transcriptional regulation occurring during early oogenesis. The complexity of the spatio-temporal expression of maternal transcripts in egg chambers allows for the exploration of a wide range of mechanisms that are crucial to the life cycle of mRNAs.

## Introduction

Spatio-temporal timing of mRNA translation is critical across eukaryotic life. Precise translational silencing and localization of mRNAs are highly dynamic processes orchestrated by nuclear and cytoplasmic proteins which are recruited to the transcripts to form mRNP granules. Forming phase-separated environments within the cytoplasm, these granules lead to translational repression while still permitting transport of mRNAs to specific regions. With its highly polarized organization, the *Drosophila* egg chamber provides an optimal inventory for the study of these dynamic and fundamental mRNA pathways.

## *Drosophila melanogaster* Oogenesis

**Figure 1.**
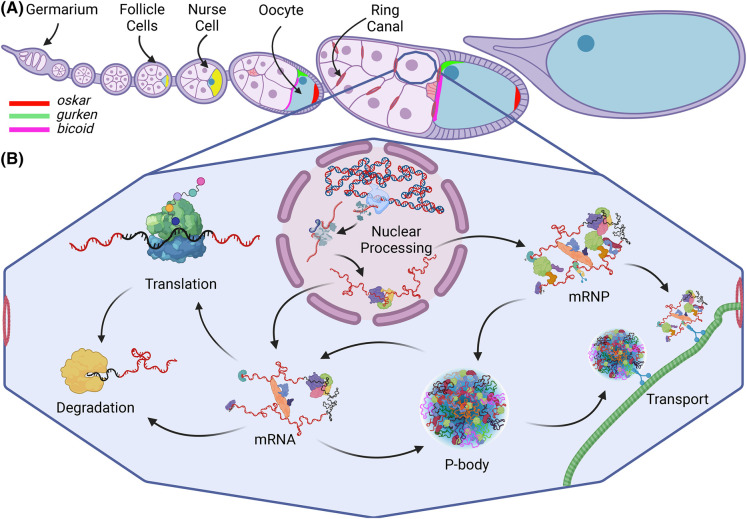
Schematic of *Drosophila melanogaster* egg chamber development and mRNA life cycle. (**A**) Ovariole chain of developing egg chambers depicting the pattern of localization in the oocyte of three key transcripts (*oskar*, *gurken* and *bicoid*) throughout oogenesis. Yellow represents the presence of all three mRNAs. (**B**) mRNA life cycle within a nurse cell. Created with BioRender.com.

The female *Drosophila* germline comprises chains of multicellular egg chambers that develop through 14 stages. Egg chamber development begins at the anterior tip of the ovariole in the germarium, where germline stem cells undergo an initial asymmetric division to yield a new stem cell and a cystoblast. After four incomplete cell divisions, the cystoblast yields a germline cyst of 16 cells. One becomes the future oocyte while the other 15 develop into nurse cells. All 16 are linked by cytoplasmic bridges (ring canals) and are encapsulated by a monolayer of somatic follicle cells (Figure 1). The predominantly transcriptionally inactive oocyte receives from the nurse cells maternal mRNAs and proteins required for its future development (reviewed in [1,2]). Maternal mRNAs, therefore, relocate across significant distances via the cytoskeletal network to reach the oocyte. During their molecular voyage these mRNAs remain structurally intact and translationally repressed until cued for translation within the oocyte. Being large and transparent, egg chambers lend themselves to visualization via molecular probes and advanced microscopy, making them a uniquely popular and successful model for the study of post-transcriptional control.

## Maternal mRNAs

A host of maternally deposited mRNAs must be localized and translated in the oocyte at precisely timed developmental stages to ensure proper embryogenesis. Coordination of this complex spatio-temporal regulation requires multiple levels of transcript-centered self-regulation throughout the mRNA's life cycle (Figure 1). Within the nucleus, primary mRNA sequences (*cis-*elements) mediate the direct binding of *trans-*acting RNA-binding proteins (RBPs) to promote nuclear egress and dictate the fate of each mRNA. Once in the cytoplasm, mRNA primary sequences and secondary structures recruit additional protein factors to generate unique mRNP macromolecular complexes. Many mRNAs are subsequently translated by ribosomes, while others are deposited at specialized cytoplasmic regions in translationally repressed states [3] (Figure 1).

Four crucial maternal transcripts that form mRNPs — *oskar* (*osk*), *bicoid* (*bcd*), *gurken* (*grk*), and *nanos* (*nos*) — have played important roles in enhancing our understanding of post-transcriptional gene regulation. For example, *grk* and its locally expressed protein Grk help establish the oocyte's body axes. Grk first influences the anterior-posterior axis (stages 5 and 6) followed by the dorsal-ventral axis (stage 7) [4]. *Osk* and *nos* mRNAs localize at the posterior pole while *bcd* mRNA is anchored at the oocyte's anterior cortex. These mRNAs remain repressed until they are translated during late stages of oogenesis (*osk/nos*) or early embryogenesis (*bcd*). Osk initiates future germline formation and, together with Nos and Bcd, establishes the embryonic anterior-posterior axis (reviewed in [5,6]). With over 250 publications on its regulation, *osk* mRNA is one of the most extensively studied and well-understood models of post-transcriptional regulation. Therefore, we selected it to serve as our case study for exploring various complex events occurring throughout the mRNA life cycle (Figure 1).

## mRNA life cycle

### Nuclear processing

**Figure 2.**
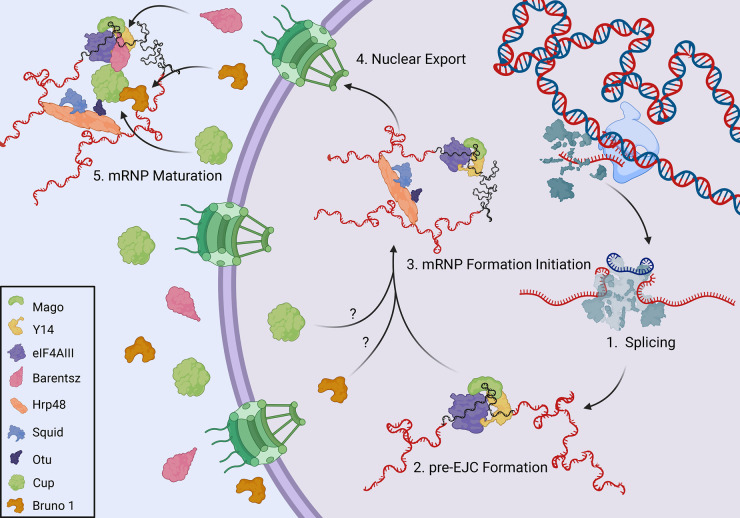
Nuclear processing and export of *osk* mRNA. Created with BioRender.com.

The ultimate fate of mRNA and the pathway it follows are inherent in the RNA primary sequence and are established co-transcriptionally through interactions with *trans*-acting nuclear proteins. This is exemplified by the formation of *osk* mRNP, which begins with the recruitment of the pre-Exon Junction Complex (EJC), consisting of eIF4AIII, Mago nashi (Mago), and Y14 to the transcript [7–9] (Figure 2). The presence of this complex at the junction of exons 1 and 2, upon proper splicing of intron 1, contributes to downstream events within the cytoplasm [10,11]. Hrp48 also joins *osk* mRNP, simultaneously binding both the 5′ and 3′ untranslated regions (UTR), thus contributing to the circularization of the transcript [12]. Hrp48 associates with nuclear proteins Otu and Squid, and together are important for the transport and localization of *osk* and *grk* mRNAs into the oocyte, reflecting a common step in these transcripts’ regulation. However, how these proteins are involved in this process is yet to be elucidated [13–15]. EJC formation is complete upon Barentsz binding at the cytoplasmic perinuclear region, which in turn recruits the motor protein Kinesin-1 to *osk* mRNP [16,17] (Figure 2).

**Figure 3.**
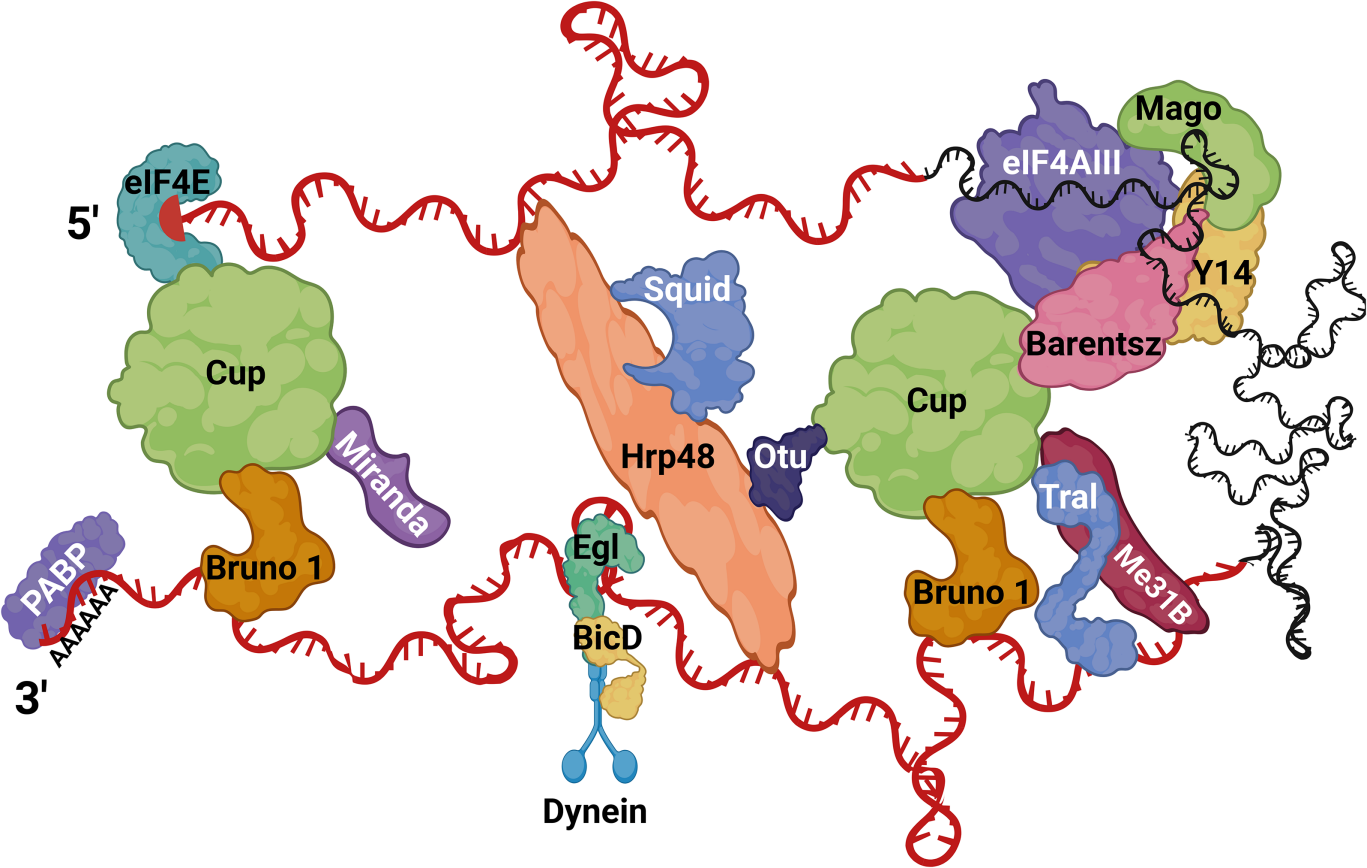
Hypothetical model of cytoplasmic *osk* mRNP. A simplified translationally repressed *osk* mRNP is represented, where a subset of protein factors (not encompassing all of the literature) are recruited to different regions of the transcript, along with several factors binding in *trans*. 5′- and 3′- UTRs are depicted in red, and the coding region in black. Created with BioRender.com.

Cup is a key protein factor in the translational control of *osk* mRNA. Initial studies found that Cup genetically interacts with the Hrp48/Otu/Squid complex [15,18]. Furthermore, Cup localizes to the nucleus in tissue culture, and interacts with the nuclear pore complex protein Nup154, raising the possibility that it may be recruited to the complex as early as nuclear processing, perhaps initiating translational repression upon mRNA export [19,20]. Nevertheless, a recent study demonstrated that Cup is capable of direct mRNA binding, but whether Cup binds *osk* mRNA directly via its intrinsically disordered regions remains uncertain [21]. Moreover, proteins eIF4E and Bruno 1 may also recruit Cup to the *osk* mRNP [22]. As an eukaryotic translation initiation factor, eIF4E binds the m^7^G cap of mature mRNAs and is necessary for recruiting the translation initiation complex via binding eIF4G to facilitate cap-dependent translation [16]. Cup binds to the same site on eIF4E as eIF4G and can therefore inhibit the initiation of translation. Cup also directly binds Bruno 1 which is recruited to *osk*’s 3′-UTR via two Bruno Response Elements [23] (Figure 3). Both Bruno 1 and eIF4E directly bind Cup and lead to the formation of a translationally repressed mRNP. Our recent work suggests this complex may first assemble in the nucleus, an observation that needs further confirmation [24]. It has also been shown that Cup recruits Barentsz to *osk* mRNA, which would suggest a role for Cup in Kinesin-1 recruitment [16].

### Cytoplasmic processing

#### mRNP formation

mRNAs slated for a specific region of the oocyte are transcribed in nurse cell nuclei and remain translationally silent, mediated primarily via their 3′-UTR *cis-*elements that recruit a cascade of numerous RBPs which take on ever more complex arrangements (reviewed in [25,26], Figure 3). Many mRNAs recruit the same proteins to maintain their stability and translational repression. For example, Bruno 1, which contains two RNA-recognition motifs (RRM), RRM 1 + 2 and an extended RRM 3, binds not only to *osk* mRNA but also to other maternal mRNAs including *grk*, *cyclin A*, *sex lethal*, and *germ cell-less* [27]. Bruno 1 dimerization promotes the formation of large particles that may also contribute to their translational silencing [28]. Particle formation via dimerization is not exclusive to Bruno 1, as *osk* 3′ UTR contains a stem–loop structure that promotes *osk* mRNA dimerization *in vitro* and its localization via ‘hitchhiking’ *in vivo* [29].

In a similar manner, Cup interacts with a variety of proteins and plausibly can join different mRNPs. To underscore this idea, Cup is known to interact with *osk* and *grk* mRNPs via Squid and PABP55 [18]. In addition, it is involved in *cyclin A* mRNA regulation possibly through Bruno 1 binding, as well as *nos* mRNA silencing, through Smaug, conceivably aiding in its activity [30–34]. The regulation of such diverse mRNAs by the same proteins poses an intriguing problem. A reasonable explanation may be that these proteins first associate with various mRNAs in the nurse cell cytoplasm as an evolutionarily conserved means of stabilizing the efficient and robust transport into the oocyte as silenced mRNP complexes. After entering the oocyte, individual mRNPs reorganize more narrowly to guide the specific relocation and translational timing of mRNAs.

#### Biomolecular condensates

**Figure 4.**
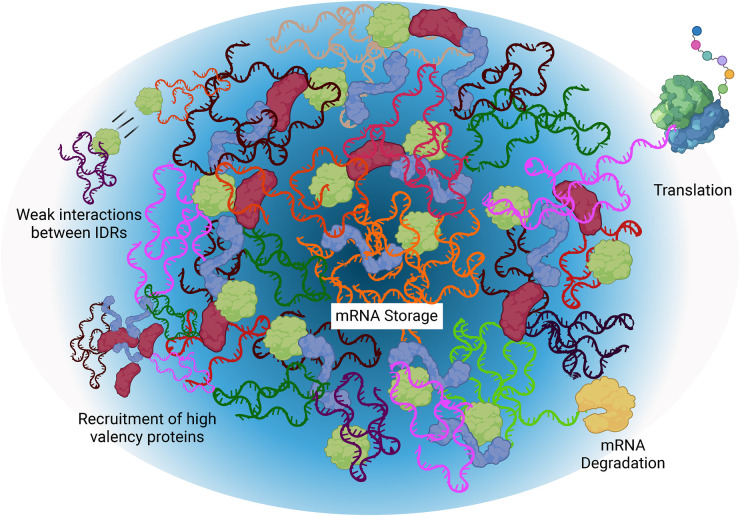
Schematic of P-body condensate and the mRNA processes it supports. Darker regions indicate the ‘core’ and lighter regions indicate the external ‘shell’. Created with BioRender.com.

Many proteins that facilitate translational repression contain intrinsically disordered regions, which makes them highly conducive to undergoing liquid-liquid phase separation (LLPS). Separation occurs when a localized concentration of proteins and mRNAs diverges sufficiently from the surrounding cytoplasm to allow for thermodynamically favorable condensation into a separate phase of mRNPs [35–38]. Condensate formation adds another layer of regulation important for translational control since it drastically changes the availability of protein binding sites [39]. Importantly, the molecular environment of the separated phase can be attenuated rapidly by local cues to allow for dynamic changes in translational repression [40] (Figure 4).

mRNAs act as super-scaffolds bringing intrinsically disordered and high-valency proteins in close proximity thus nucleating condensation events [41,42]. The ‘high valency’ classification characterizes proteins that can bind many other proteins to bring about phase separation into membraneless organelles (MLO) such as P-bodies [43]. P-bodies are cytoplasmic, non-membrane bound organelles, crucial for normal cellular functions [44]. They are devoid of ribosomes and have a semi-ordered structure (reviewed in [45]) (Figure 4). In *Caenorhabditis elegans* and *Drosophila* germ granules, mRNAs exhibit self-demixing, and show a biphasic organization, where translation occurs in the outer phase, while mRNAs housed in the inner phase are translationally repressed, a mechanism that may similarly function in *Drosophila* P-bodies [46–52]. Maternal mRNAs exhibit differential levels of association with the P-body ‘core’ and ‘shell’ regions, which have been implied to correspond with their translational fate. For example, *bcd* mRNA is translationally silenced throughout oogenesis and localizes to the core, while *grk* mRNA must be poised for two rounds of translation and localizes to the shell region [53] (Figure 4).

Many protein components of the *osk* mRNP facilitate LLPS and have been shown to participate in P-body formation, including YPS, Trailer Hitch (Tral), Exuperantia, Me31B, Hsp90, and Ge1. Not surprisingly, these proteins are also implicated in *osk* mRNA silencing and its transport [54–59]. Interestingly, the *osk* transcript itself also has non-coding roles that support oogenesis, possibly in initiating P-body formation [60]. Furthermore, nucleotide mutations in *osk* mRNA can disrupt condensate formation *in vitro*, suggesting a direct role for mRNA sequences in condensate integrity [61].

P-bodies in the *Drosophila* germline contain proteins involved in several different aspects of RNA metabolism, including mRNA decapping activators DCP1, DCP2, and Ge1, DEAD-box RNA helicase Me31B, and translation repressors Tral and Cup amongst others (reviewed in [45]). A core component of P-bodies, Me31B was first identified as a gene essential for egg chamber development [62]. Me31B is also implicated in the translational regulation of several maternal mRNAs: *osk*, *bcd*, *grk*, *bic-D*, *nos*, *orb*, *polar granule component*, and *germ cell-less* [40,53,58,63,64]. Moreover, Me31B is important for translational silencing of *osk* mRNA during its transport, with this interaction likely occuring in P-bodies [24,58].

Cross-regulation seems to be a hallmark of P-bodies, as P-body proteins have been shown to regulate the expression of other P-body mRNAs that may be mediated transcriptionally, within the P-body, or through another mechanism. One such example is Me31B, where *tral* and *cup* mRNA levels decreased in *me31B* mutant egg chambers indicating a yet-to-be-elucidated feedback loop [65,66]. A complex cycle of regulation also extends to Cup, which associates with the protein products of the mRNAs it represses. For example, it associates with Osk protein at the posterior, where Osk is necessary for the translational activation of *nos* mRNA [67]. Once translated, Cup interacts with Nos protein to promote normal development of the ovarian germline [68].

Egg chambers contain a variety of MLOs, such as the perinuclear nuage in nurse cell cytoplasm and the germ granules in the oocyte (reviewed in [69]). Most other cytoplasmic MLOs not induced by stress come under the general category of ‘P-body’. Unfortunately, this simplistic classification is misleading since P-bodies exhibit substantial heterogeneity. Their complex composition with diverse proteins determines their various functions; for example, Tral and EDC3 (enhancer of Decapping 3) compete for the same FDF pocket on Me31B, making their binding mutually exclusive. When the ratio of EDC3 is higher than Tral in the P-body, mRNA degradation is favored, as EDC3 can also recruit DCP2 (Decapping protein 2). Similarly, if the ratio of Tral is higher than EDC3, Tral can recruit DCP1 (Decapping protein 1) to the P-body and leads to degradation as well. Further convolving the picture of condensate composition, Tral binds either Cup or DCP1 at the same LSm site [70]. Thus, P-bodies that contain Cup, in turn lead to translational silencing and stable mRNA storage [70]. Cup recruits the deadenylase complex CAF1-CCR4-NOT and leads to deadenylation of mRNAs while suppressing the decay activity of CAF1-CCR4-NOT itself [71]. Shortening of poly(A) tails is a widely conserved regulatory mechanism especially for maternal mRNAs, further ensuring their translational repression (reviewed in [72]). Although comprising distinct protein arrays, these MLOs are still identified simply as P-bodies. More accurate naming would reflect the fact that condensates are molecular decision-making centers where transcripts linger stably either in ‘repression-bodies’ (‘r-body’, such as Me31B-Tral-Cup) until translated or in ‘degradation-bodies’ (‘d-body’, such as Me31B-Tral-DCP1 and Me31B-EDC3-DCP2) until destined for decay.

#### mRNP localization

Asymmetric localization of mRNA is intrinsic to many fundamental processes, including cell motility, synaptic plasticity, and organism development. In *Drosophila* embryos 71% of mRNAs were found to localize asymmetrically, implying a global cellular phenomenon [73]. *Drosophila* oogenesis is no exception. An unbiased global analysis of mRNA localization by Jambor et al. generated a comprehensive resource, the Dresden Ovary Table, that significantly expands research support into intracellular areas of mRNA concentrations [74]. Among the 3475 mRNAs they identified to be expressed during oogenesis, 35% exhibit subcellular localization. On average, localized mRNAs, interestingly but not surprisingly, carry longer 3′-UTRs that suggest a more elaborate post-transcriptional regulation. At the same time, mRNAs localized at the posterior region also carry longer 5′-UTRs, more numerous introns and exons, and are expressed at significantly higher levels. Enrichment of non-coding regions suggests that these localized mRNAs perform a function apart from translation of their protein products alone [74].

**Figure 5.**
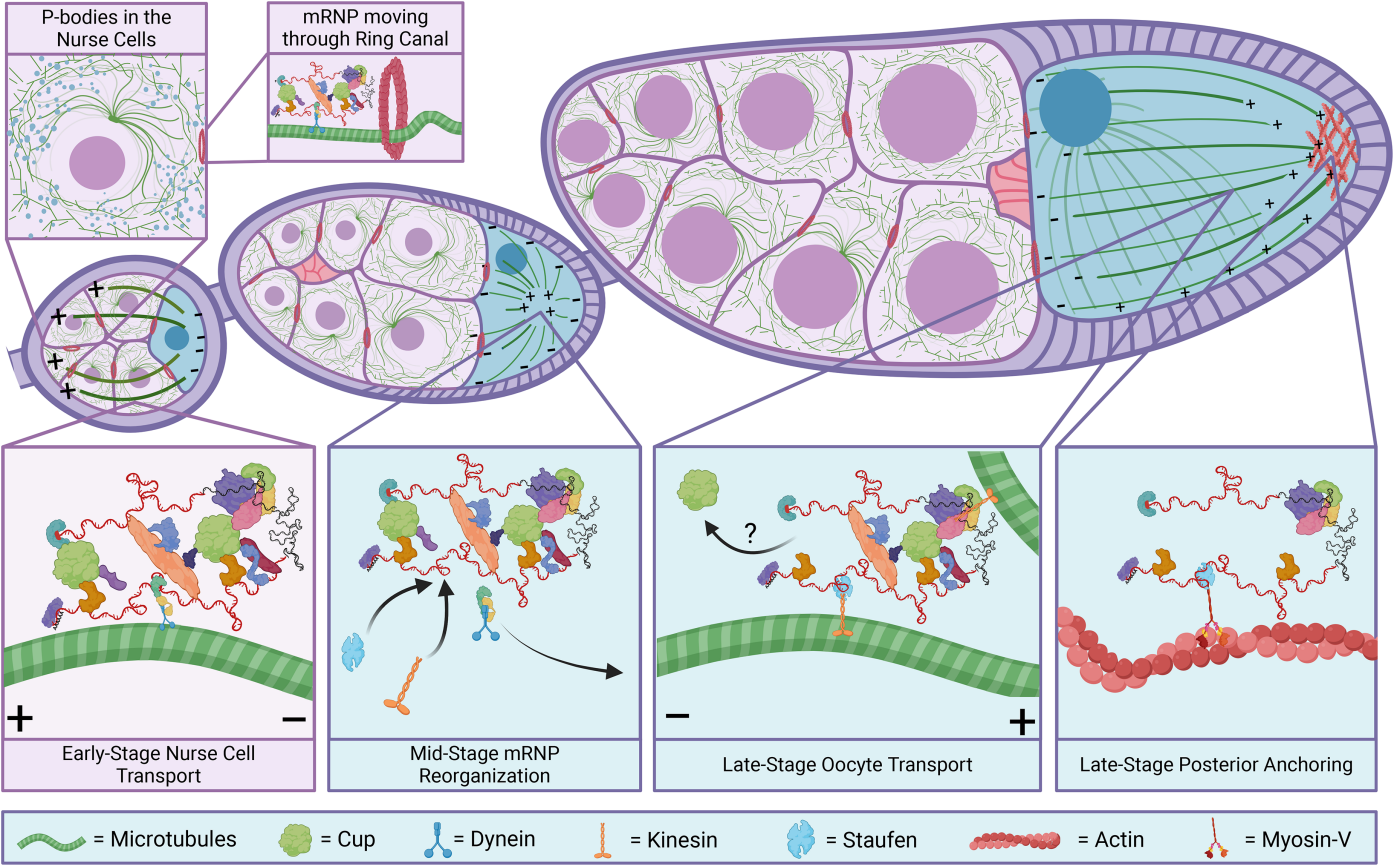
The microtubule network and mRNP transport throughout oogenesis. Created with BioRender.com.

Biological condensates provide plausible mechanisms for asymmetric mRNA localization. Since condensates contain multiple RNPs, their coupling to the microtubule network may be more energetically efficient than individual RNP transport (Figure 5). Consistent with this view is the association of the microtubular motor protein Dynein with P-bodies, hinting at a role for P-bodies in long-distance localization [75]. A recent study by Baker et al. supports this connection by showing that Egalitarian, a Dynein adaptor protein, directly interacts with Me31B [76]. *Drosophila* oogenesis serves as a model for this mode of directed transport since normal cell function requires transcript movement over considerable distances at rates greater than passive diffusion can provide. However, some mRNPs, for example *grk* and *osk*, are known to move via microtubules independently of condensates, leading some to conclude that condensates are a consequence of normal mRNA movement [77]. More likely, many mRNPs may translocate from nurse cells to the oocyte by varying means that reflect the dynamic nature of the microtubule network.

During stages 2–6 of egg chamber development, the microtubular network is arranged with its minus (−) ends within the oocyte while the plus (+) ends extend into the nurse cells (Figure 5). Transcripts therefore associate with (−)-directed motor proteins to enter the oocyte. At stage 6, signals from Grk to the somatic follicle cells cause the microtubules to reorganize, orienting the microtubule (+) ends toward the center of the oocyte. Starting at stage 8, localized inhibition of PAR-1 kinase diminishes microtubule nucleation at the oocyte posterior resulting in the redirection of microtubule (+) ends toward the posterior egg chamber for the remainder of late oogenesis, and concludes with nurse cell dumping and ooplasmic streaming [78,79].

Localization of mRNAs relies on *cis-*elements in the mRNA (mostly in the 3′-UTR) bound by *trans-*acting RBPs that can further recruit other factors in fine-tuning mRNA regulation (reviewed in [80,81]). These mRNPs can self-regulate their own dynamic participation in active transport via motor proteins on microtubules, along with passive transport by cytoplasmic streaming and/or localized degradation. Localizing elements in the transcript can take a variety of forms; for example, primary sequences or complex secondary structures that act as destination zip codes within the cytoplasm (reviewed in [3,82–84]). Yet, these sequences alone do not fully explain mRNA localization; Jambor et al. have demonstrated differential localization at various developmental stages even without changes in the primary sequence [74].

How cells resolve this divergent localization continues to be a perplexing process, but the emerging picture indicates that an exchange of mRNP factors must be involved. Once again, *osk* mRNA serves as an example: its transport from nurse cells to the oocyte until stage 6 is facilitated mainly by the Bicaudal-D/Egalitarian/Dynein complex that distributes *osk* mRNA into the oocyte [77,85–87]. Microtubules rearrange during stages 7 and 8, and *osk* mRNA concentrates in the middle of the oocyte. By stage 9, microtubule (+) ends become slightly enriched at the posterior of the oocyte, thereby ensuring that *osk* mRNA, now transported by Kinesin-1, reaches its proper location [88,89] (Figure 5).

Staufen, a double-stranded RBP, mediates the switch from Dynein to Kinesin-1 and is responsible for *osk* mRNA localization to the posterior cortex of the oocyte [90,91]. Staufen associates with the complex only after the mRNP reaches the oocyte and the microtubules undergo reorganization [92]. After Staufen joins the complex, Egalitarian dissociates from the mRNP to allow for the switch to Kinesin-1 [91,93]. Once the complex reaches the posterior of the oocyte, an actin motor protein, Myosin-V, mediates *osk* mRNA anchoring by out-competing Kinesin-1 due to the low concentration of microtubules [94,95]. Similar competition between two motor proteins was observed in neurons [96]. These molecular orchestrations ensure proper cortical localization of *osk* mRNA along with its anchoring at the posterior cortex and persistent maintenance by the long Osk isoform to actin filaments [94].

By contrast, at the oocyte's anterior half, a higher density of microtubules favors Staufen binding followed by the transport of *osk* mRNA toward the posterior of the oocyte [92,94,97]. Staufen is also involved in the anterior localization of *bcd* mRNA [98]. How Staufen accomplishes localization of *bcd* and *osk* mRNAs to opposite ends of the oocyte remains an enigma. Different mRNP components, for example Miranda, may point to an explanation for this juxtaposition of roles. Miranda-GFP expression in the female germline leads to an ectopic localization of Staufen/*osk* mRNA complex at the anterior due to coupling between Staufen/*osk* and the *bcd* mRNA localization pathway [99]. Compellingly, Cup directly interacts with Miranda and also associates with Staufen at the same sites where Miranda/Staufen associates, leading to a complex, hypothetical model where Miranda cannot bind to Staufen in the presence of Cup, thereby allowing correct localization of *osk* mRNA to the posterior [100]. It would be interesting to determine whether egg chambers that express Miranda-GFP also exhibit reduced colocalization of Cup with *osk* mRNA. A second plausible explanation involves Exuperantia, which is necessary for the anterior localization of *bcd* mRNA, but it is not thought to be a member of the *osk* mRNP. In addition, *bcd* mRNA anchoring in the anterior depends on Dynein and is independent of the oocyte's microtubule network [101–103].

Further underscoring this conceptualization, the Bono group determined that there is a highly complex network of six evolutionarily conserved RBPs (Staufen, Vasa, eIF4AIII, Nanos, Hrp48 and Glorund) that are crucial for the regulation of the four patterning mRNAs [104]. Their research illuminates the complex interplay between these RBPs and how they collectively orchestrate the differential cytoplasmic fates of mRNAs. For instance, while Cup is a crucial component of both *grk* and *osk* mRNPs, Staufen is only part of *osk* mRNP [16–18,92,97]. This exemplifies the regulation of *grk* and *osk* mRNAs to involve unique sets of RBPs and suggests that the differential functions of these mRNPs resides within the distinct selection and incorporation of various factors.

#### Translation

Once the mRNP complex reaches its destination, translational de-repression is triggered by mechanisms that remain poorly understood during oogenesis. The silencing factors must be removed and the mRNP undergoes reorganization. This mechanism is better grasped in the early embryo, where during the maternal-to-zygotic transition, an influx of signals leads to the degradation of P-body proteins and a general release from translational repression of the transcripts contained in the condensate [105]. This level of regulation means that a wide array of transcripts can be readily released simultaneously from translational repression. A caveat to this mechanism is that alterations in the cellular environment, such as changes in salt concentration or ATP depletion, can result in the hardening or dissolution of condensates, thereby impacting the proper post-transcriptional regulation of mRNA and leading to pathological outcomes [106,107].

## Perspectives

- Translational control is a complex process epitomized in the *Drosophila melanogaster* germline where maternal mRNAs are precisely and dynamically regulated at the post-transcriptional level. Not surprisingly, many aspects of RNA processing, including RNA export, localization and translational repression, are now known to be dysregulated in, and contribute to, numerous diseases, such as neurodegeneration, cancer and infectious diseases [108,109]. MLO continue to emerge in disease etiology, thus, it is important to understand the basic mechanisms governing post-transcriptional control and how these processes are converted to drive disease. *D. melanogaster* egg chambers provide an exceptional system for studying such aspects of basic mRNA biology. Their genetic malleability, together with numerous genetic toolkits as well as the ease of manipulation of a developing multicellular system, allow for specialized techniques such as super-resolution visualization and *in vivo* biochemical interactome assays, making them ideal to carry out such research.
- RNP granules play key roles in organizing spatial and temporal regulation of RNA-dependent processes. The diversity is supplied by the dynamic exchanges of *trans*-acting proteins that are orchestrated throughout an mRNA's life cycle. Cytoplasmic organelles without membranes are now center-stage players in cell biology, especially where multifaceted mRNA regulation (localization, translation, and stability) occurs. The *D. melanogaster* egg chamber serves as a model for long-range mRNA cytoplasmic transport and presents an innovative vehicle to enhance our understanding of both mRNP cellular transport and interactions with P-bodies.
- We have much to learn about the communication between the layers of mRNA regulation and the precise mechanisms underlying translational control. As technological and imaging advances continue to enlighten the world of molecular and cellular biology, new details of mRNA dynamics become unveiled. In contrast to cultured cells, *D. melanogaster* egg chambers contain substantially more P-bodies, which are readily visualized and can be chemically and genetically manipulated. These unique characteristics position them ideally to resolve some of the most persistent and outstanding questions in mRNA biology at the post-transcriptional level. Future work in polarized tissues can provide an important insight into MLOs' biological functions, which are much debated and still poorly understood. Deciphering how cells manage mRNA regulation in P-bodies will, therefore, be crucial to improve the management of pathological processes such as cancer progression, chemo-resistance and viral infections [45,110,111].

## Abbreviations

LLPS: liquid-liquid phase separation
MLO: membrane-less organelles
RBP: RNA-binding protein
RRM: RNA-recognition motifs
UTR: untranslated regions
